# Disaggregation of Ploidy, Gender, and Genotype Effects on Wood and Fiber Traits in a Diploid and Triploid Hybrid Poplar Family

**DOI:** 10.3389/fpls.2022.866296

**Published:** 2022-04-01

**Authors:** Xu-Yan Huang, Jing Shang, Yu-Hang Zhong, Dai-Li Li, Lian-Jun Song, Jun Wang

**Affiliations:** ^1^National Engineering Research Center of Tree Breeding and Ecological Restoration, Beijing Forestry University, Beijing, China; ^2^Key Laboratory of Genetics and Breeding in Forest Trees and Ornamental Plants, Ministry of Education, Beijing Forestry University, Beijing, China; ^3^The Tree and Ornamental Plant Breeding and Biotechnology Laboratory of National Forestry and Grassland Administration, Beijing Forestry University, Beijing, China; ^4^College of Biological Sciences and Technology, Beijing Forestry University, Beijing, China; ^5^Beijing Institute of Landscape Architecture, Beijing, China; ^6^Breeding and Propagation Base for Tree Varieties in Weixian County, Xingtai, China

**Keywords:** *Populus*, allotriploid, gender, genotype, wood property, fiber traits, triploid breeding

## Abstract

Triploid breeding based on unilateral sexual polyploidization is an effective approach for genetic improvement of *Populus*, which can integrate heterosis and ploidy vigor in an elite variety. However, the phenotypic divergence of unselected allotriploids with the same cross-combination remains poorly understood, and the contributions of ploidy, gender, and genotype effects on phenotypic variation are still unclear. In this study, wood and fiber traits, including basic density (BD), lignin content (LC), fiber length (FL), fiber width (FW), and fiber length/width (FL/W), were measured based on a 10-year-old clonal trial, including full-sib diploid and triploid hybrids of (*Populus pseudo-simonii* × *P. nigra* ‘Zheyin3#’) × *P.* × *beijingensis*, and contributions of ploidy, gender, and genotype effects on the variation of these traits, were disaggregated to enhance our understanding of triploid breeding. We found a significant phenotypic variation for all measured traits among genotypes. All the wood and fiber traits studied here underwent strong clonal responses with high repeatabilities (0.55–0.76). The Pearson’s correlation analyses based on the best linear unbiased predictors (BLUPs) revealed that BD was significantly positively correlated with FL (*r* = 0.65, *p* = 0.030), suggesting that BD could be improved together with FL during triploid breeding. The FL of the triploids was significantly larger than that of the diploids (*p* < 0.001), suggesting that ploidy strongly affected the variation of FL traits. The difference between females and males was not significant for any measured trait, implying that gender might not be a major factor for variation in these traits. Further analyses of variance components showed that genotype dominantly contributed to the variation of BD, LC, and FW traits (with 54, 62, and 53% contributions, respectively) and ploidy contributed strongly to variation in FL and FL/W (77 and 50%, respectively). The genetic coefficient of variation (CV_G_) of triploids for each trait was low, suggesting that it is necessary to produce many triploids for selection or to use different *Populus* species as parents. Our findings provide new insights into the genetic effects of ploidy, gender, and genotype on wood and fiber traits within a full-sib poplar family, enhancing the understanding of the triploid breeding program of *Populus*.

## Introduction

The species in the genus *Populus*, characterized by fast growth, good wood properties, strong resistance, and wide adaptability, are widely distributed over the northern hemisphere (Porth and El-Kassaby, 2015; Georgii et al., 2019). They are used not only in urban landscaping, ecological remediation, and rehabilitation of degraded lands, but also for wood and fiber production (Bannoud and Bellini, 2021). As an important source of industrial raw timber, an improvement on wood properties of *Populus* is a major focus of breeders. Wood and fiber properties, such as basic density (BD), lignin content (LC), and fiber length (FL), directly affect pulp value and paper quality.

Triploid breeding is an effective approach for the genetic improvement of tree species, which can achieve multitarget trait improvement on growth, wood properties, and stress resistance (Kang, 2020). In *Acacia*, allotriploid clones derived from crossing tetraploid *Acacia mangium* with diploid *A. auriculiformis* possessed a higher heartwood proportion and bark thickness than commercial diploid clones (Bon et al., 2020). Serapiglia et al. (2014, 2015) found that triploid genotypes of shrub willow could produce more biomass and possess lower LC than diploid genotypes. In *Populus*, some triploid varieties have been widely used for plantations, all of which exhibited favorable growth and pulpwood characteristics (van Buijtenen et al., 1958; Weisgerber et al., 1980; Zhu et al., 1995; Kang, 2016).

Unilateral sexual polyploidization based on the hybridization of uniparental 2n gametes is the main way for triploid breeding of *Populus* (Kang, 2016). Sexual polyploidization, integrating the contributions of hybridity and genome dosage, can cause extensive phenotypic variation (Fort et al., 2016). In *Populus*, therefore, understanding trait variation associated with triploidy is of considerable interest to breeders. Zhu (2006) indicated that not all triploid individuals of *Populus* are elite, so triploid breeding should follow the strategy of strong selection in a large candidate population. In hybrids of (*P. alba* × *P. glandulosa*) × *P. tomentosa*, although the 2-year seedling height and ground diameter of 12 triploids were 33 and 38% larger than those of the diploids on average, respectively, several triploid genotypes exhibited lower growth performance than the diploids (Li and Kang, 2007). Wu et al. (2013) found that the elite triploid genotypes of *P. tomentosa* hybrids had favorable wood density and fiber traits. However, the variation in wood and fiber traits of unselected allotriploids within the same cross-combination was considerable, and the contributions of ploidy and genotype on the variation of wood properties were not clarified.

As dioecious trees, members of the genus *Populus* provide opportunities to study the relationship between trait variation and sexual dimorphism in perennial woody plants. In a recent review, Melnikova et al. (2017) reviewed the sex-specific response to the stress of *Populus*, which demonstrated that the males of the *Populus* species were better adapted to the stress conditions and showed less damage, better growth, and higher photosynthetic capacity and antioxidant activity than that of the females. In *P. purdomii*, it was found that the males had a quicker energy-return strategy in high-altitude areas (Lei et al., 2016). For wood properties, in a collected natural diploid population of *P. tomentosa*, the females had a significantly larger FL and fiber width (FW) than the males on average (Du et al., 2014). However, in allotriploids, the effect of gender on wood and fiber traits remains poorly understood.

In our previous work, a full-sib family including triploid hybrids derived from crossing between induced 2n eggs of *Populus pseudo-simonii* × *P. nigra* ‘Zheyin3#’ and normal pollen of *P.* × *beijingensis* and diploid hybrids of the two parents were obtained (Wang et al., 2010). After cutting propagation, the diploid and triploid hybrids were planted in the trial field of Weixian County, Hebei Province, China, in 2010. In this study, the wood and fiber traits, including BD, LC, FL, FW, and fiber length/width (FL/W), were measured in the diploid and triploid hybrids, and contributions of ploidy, gender, and genotype effects on the variation of the wood and fiber traits were disaggregated. This enhanced our understanding of trait variation resulting from sexual polyploidization and provided additional data for the selection of elite genotypes.

## Materials and Methods

### Plant Material

Nine diploid hybrids and 11 triploid hybrids between female parent *Populus pseudo-simonii* × *P. nigra* ‘Zheyin3#’ (2n = 2x = 38) and male parent *P.* × *beijingensis* (2n = 2x = 38, abbreviated as BJY) were analyzed in this study. The triploid hybrids were derived from crossing induced 2n eggs through colchicine-induced embryo sac chromosome doubling of ‘Zheyin3#’ with the BJY (Wang et al., 2010). All the 2n eggs were determined as post-meiotic restitution (PMR) type 2n gametes by simple sequence repeat (SSR) analysis (Dong et al., 2015). After cutting propagation, a clonal trial was established in 2010 at the Weixian County, Hebei Province, China, based on a randomized complete block design with three blocks and four ramets per plot with 3 m × 4 m tree spacing. The trial was irrigated for six times in total, in March, May, and August of the first 2 years, with no subsequent irrigation. Weed control was carried out in the first 3 years. No fertilizer was used in the field. No thinning was applied during the trial period until the time of sampling. Gender identification was carried out after flowering. In the 20 genotypes, there were 4 female diploid genotypes, 5 male diploid genotypes, 5 female triploid genotypes, and 6 male triploid genotypes. In 2019, wood samples were collected from the clonal trial. A number of three ramets (one per plot) were randomly selected from each clone for sampling. A 10-cm-thick wood disk at breast height (1.3 m) was taken from each sample tree. In total, 60 disks were harvested for laboratory measurement.

### Measurement of Wood and Fiber Traits

For BD analysis, four small rectangular pith-to-bark direction wood specimens with dimensions of 40 mm radially × 20 mm tangentially × 20 mm longitudinally were cut from each disk at four directions. The seventh annual ring was located at the middle of each specimen in the radial direction. The BD was analyzed based on the maximum moisture content method described by Smith (1954).

For intra-ring analysis of fiber properties, four matchstick-sized wood specimens were excised from the above rectangular wood specimens at the seventh and eighth rings and then macerated in a 1:1 (*v*/*v*) mixture of acetic acid and hydrogen peroxide at 60°C for 24 h. After rinsing three times with distilled water, the specimens were vibrated to scatter fibers in test tubes. Then, the fibers were stained with safranin solution and observed under an Olympus BX51 microscope. The FL and FW were measured using an ocular micrometer. More than 200 fibers were measured for each specimen. FL/W was calculated based on the FL and FW values.

The LC was measured following the standard procedure for biomass analysis developed by the National Renewable Energy Laboratory (NREL) of the United States (Sluiter et al., 2008). The LC was equal to the sum of acid-soluble LC and acid-insoluble LC. Each sample was analyzed in triplicate.

### Data Statistical Analysis

Statistical analyses were performed in the R statistical environment (R Development Core Team, 2007). The results are presented in this study as mean ± SE. One-way analysis of variance (ANOVA) was performed on each trait, and the means were compared using a protected least significant difference (LSD, *p* < 0.05) to reveal the difference among genotypes. The repeatability (*R*^2^) of each measured trait was estimated using the repeatability function in the heritability R package (version 1.3) developed by Kruijer et al. (2015). Best linear unbiased predictors (BLUPs) of each genotype, genetic coefficient of variation (CV_G_), and variance components of ploidy, gender, and genotype effects were estimated by the AFEchidna R package (version 1.54) developed by Zhang et al. (2021) with model: trait ∼ 1 + ploidy + ploidy:gender + ploidy:gender: genotype. Variance contributions were calculated based on the variance components of ploidy, gender, and genotype effects, and residuals were considered as the results of environmental effects. Pearson’s correlation tests were run to analyze the relationship between all trait combinations using the phenotypic data and BLUPs, respectively. Student’s *t*-test were used to identify the statistical differences between diploid and triploid groups and between female and male groups nested in the two ploidy levels for all traits.

## Results

### Basic Statistics and Variation Among Genotypes

For the full-sib diploid and triploid hybrid poplar family, the BD ranged from 320.21 to 410.35 kg m^–3^, with an average of 355.90 ± 5.57 kg m^–3^. The LC ranged between 25.68 and 30.33% with an average of 27.62 ± 0.24%. The FL ranged from 0.890 to 1.293 mm, with an average of 1.115 ± 0.029 mm. The FW ranged from 19.05 to 26.30 μm with an average of 22.97 ± 0.40 μm. The FL/W ranged from 41.55 to 61.53 with an average of 50.05 ± 1.29. The CV_G_ of the traits in this family was 7, 4, 44, 7, and 10% for BD, LC, FL, FW, and FL/W, respectively. The CV_G_ of BD, LC, FL, FW, and FL/W in the diploids was 7, 4, 6, 8, and 6%, respectively. The CV_G_ of these traits in the triploids was 5, 3, 3, 6, and 6%, respectively.

**FIGURE 1:**
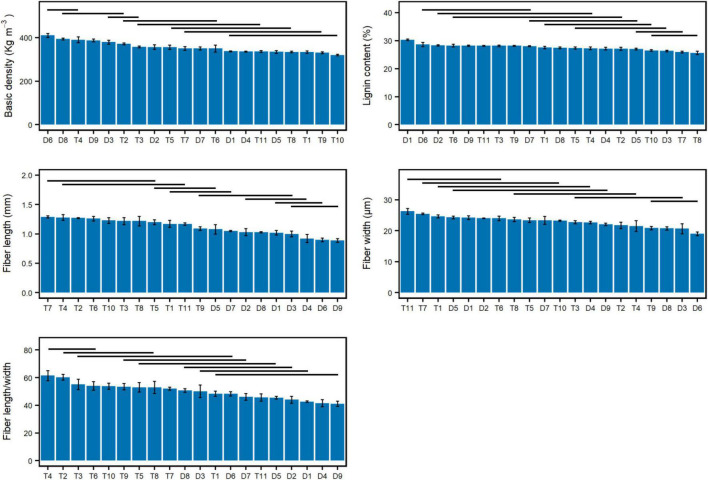
Comparison among the genotypes for the measured wood and fiber traits. “—” covers the genotypes without a significant difference at the 0.05 level.

One-way ANOVA tests revealed significant differences among the genotypes in all measured traits (Figure 1), suggesting that genotypes affected the phenotypes of wood and fiber traits. For BD, the diploid genotypes D6 and D8 and triploid genotype T4 were significantly larger than the other genotypes. The diploid genotype D1 had the highest LC. Eight triploid genotypes, including T7, T4, T2, T6, T10, T3, T8, and T5, had longer FL, which suggests that the increased ploidy level contributed to the FL trait significantly. Three diploid genotypes (D5, D1, and D2) and four triploid genotypes (T11, T7, T1, and T6) had wider FW compared with the other genotypes. The FL/W of T4, T2, T3, and T6 was statistically equal and larger than those of the other genotypes.

### Repeatabilities of Wood and Fiber Traits

**FIGURE 2:**
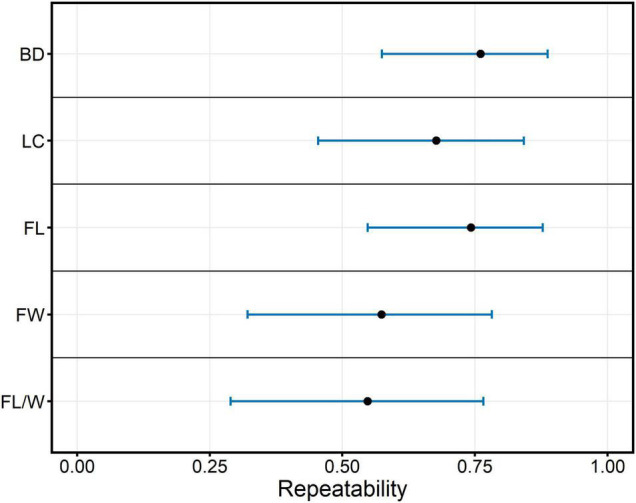
Repeatability estimates (black dots) and 95% confidence intervals (blue bars) for wood and fiber properties. BD, Basic density; LC, Lignin content; FL, Fiber length; FW, Fiber width; FL/W, Fiber length/width.

Repeatabilities (*R*^2^) for all measured traits were estimated and are shown in Figure 2. *R*^2^ of the wood and fiber properties in the whole family ranged from 0.55 to 0.76, which suggests that the observed phenotypic variation of these traits in the full-sib family was strongly affected by genetic effects. BD had the highest estimated repeatability (*R*^2^ = 0.76) with 95% confidence intervals ranging from 0.57 to 0.89. FL/W had the lowest estimated repeatability (*R*^2^ = 0.55) with 95% confidence intervals ranging from 0.29 to 0.77.

### Pearson’s Correlation Between Traits

**FIGURE 3:**
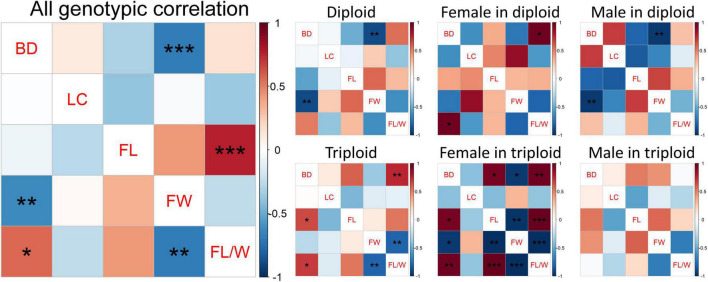
Pearson’s correlation between wood and fiber traits. The upper and lower diagonals represent phenotypic and BLUP correlations, respectively. BD, Basic density; LC, Lignin content; FL, Fiber length; FW, Fiber width; FL/W, Fiber length/width. *, **, and *** represent significance at 0.05, 0.01, and 0.001 levels, respectively.

Pearson’s correlations of both phenotype and BLUPs among all measured wood and fiber traits were calculated and are presented in Figure 3. BLUPs estimate the strength of genetic correlation between traits, which is valuable to guide plant breeding. The phenotypic correlation coefficients (*r*) between traits in the whole family ranged from −0.69 to 0.77, and the BLUP correlation coefficients ranged from −0.68 to 0.56. BD was significantly negatively correlated with FW in both phenotypic (*r* = −0.69, *p* < 0.001) and BLUP (*r* = −0.64, *p* = 0.002) levels. There was a strongly positive phenotypic correlation between FL and FL/W (*r* = 0.77, *p* < 0.001), but their BLUP correlation was not significant. Although the phenotypic correlations between BD and FL/W and between FW and FL/W were not significant, their BLUP correlations reached a significant level.

In the diploid group, BD was significantly negatively correlated with FW at both the phenotypic and BLUP levels. There were also significant negative correlations between BD and FW in diploid males. In diploid females, BD was significantly positively correlated with FL/W at both the phenotypic and BLUP levels. In the triploid group, both phenotypic and BLUP correlations between BD and FL/W and between FW and FL/W were significant, and the BD was significantly positively correlated with FL at the BLUP level. In triploid females, BD was significantly correlated with FL, FW, and FL/W. FL was strongly negatively correlated with FW and positively correlated with FL/W, and FW was strongly negatively correlated with FL/W. However, in triploid males, there was no significant correlation between the traits no matter at phenotypic or BLUP level.

### Disaggregation of Genetic Effects

**FIGURE 4:**
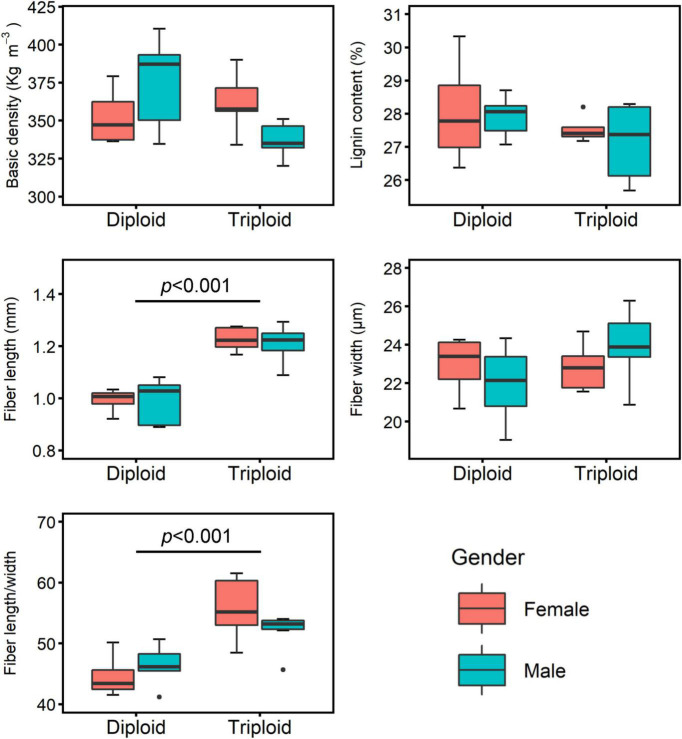
Comparisons between diploid and triploid groups and between female and male groups within the ploidy levels for wood and fiber traits.

The phenotypes of the BD, LC, FL, FW, and FL/W traits of the diploid genotypes ranged from 334.66 to 410.35 kg m^–3^, 26.37–30.33%, 0.890–1.081 mm, 19.05–24.35 μm, and 41.18–50.69, respectively. These traits of the triploid genotypes ranged from 320.21 to 390.06 kg m^–3^, 25.68–28.29%, 1.089–1.293 mm, 20.88–26.30 μm, and 45.70–61.53, respectively. The Student’s *t*-test showed that there were no statistical differences between the diploid and triploid groups on average for BD, LC, and FW traits (*p* = 0.157, 0.187, and 0.204, respectively). However, the FL and FL/W traits of the triploid group were significantly larger than those of the diploid group on average (Figure 4), which suggests that the increased ploidy level might be one of the main sources of variation for the FL and FL/W traits. Concerning the aspect of gender nested in the ploidy levels, however, no significant difference was found between females and males (*p*-values at 0.055–0.951), which suggests that the gender effect might not be a major factor for variation of the measured traits.

**FIGURE 5:**
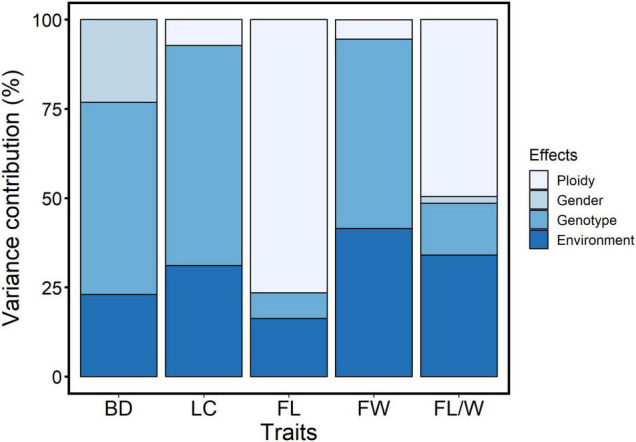
Disaggregation of ploidy, gender, and genotype effects on woody and fiber properties based on variance contributions. BD, Basic density; LC, Lignin content; FL, Fiber length; FW, Fiber width; FL/W, Fiber length/width.

Variance contributions of genotype, ploidy, and gender effects for the wood and fiber traits were analyzed and are presented in Figure 5. Genotype was the major contributor to variation in BD, LC, and FW, with 54, 62, and 53% contributions, respectively. The variations in the FL and FL/W traits were mainly attributed to ploidy (77 and 50%, respectively). The gender effect nearly did not contribute to the variation of these traits, except for the BD, which was attributed to the gender effect with 23% contribution. The environmental effects on these traits ranged from 16 to 41%.

## Discussion

Genus *Populus* is an important source of industrial raw timber in the northern hemisphere. In China, the lumber of *Populus* is widely used to produce pulpwood. The basic wood density, LC, and FL affect the pulp yield and paper quality (Kenney et al., 1990). Therefore, the improvement of pulpwood characteristics has attracted much attention. Triploid breeding has proved to be an effective way to improve the growth, wood properties, and abiotic stress of *Populus* (Kang, 2016). In white poplar, however, it was found that the BD of triploids was lower than that of the diploids (Kang, 2016). The low BD could decrease the yield of the pulp. In the triploid breeding program of the *Populus*, the selection of elite genotypes with high BD, low LC, and large FL are our key breeding objectives.

Polyploidization plays an important role in plant speciation and evolution and contributes to plant breeding (Van de Peer et al., 2009; De Storme and Mason, 2014; Sattler et al., 2016). According to the genomic composition, polyploid can be classified into two basic types: auto- and allopolyploid. In allopolyploids, phenotypic variation is commonly affected by both genomic heterozygosity and polyploidy. In *Arabidopsis thaliana*, the plant size of allotriploids was related to their parental genome dosage, but the tetraploid plants showed a low growth rate, suggesting that both the genotype and ploidy level contributed to variation in the plant size (Fort et al., 2016). In the present study, we took advantage of a full-sib progeny, including diploid and triploid genotypes, to provide new insights into the genetic effects of ploidy, gender, and genotype on the wood and fiber traits of *Populus*. All the wood and fiber traits studied here showed high repeatabilities (0.55–0.76). Genotype and ploidy were the main contributors to trait variation, suggesting that integration of heterosis and ploidy vigor is important for polyploid breeding. However, because the number of induced triploid hybrids is limited, and from a single parental combination, it is not known how generalizable these findings are.

An increased genome dosage is thought to cause many phenotypes associated with polyploidy, such as increased cell size, improved biomass yield, and enhanced production of secondary metabolites (Dhawan and Lavania, 1996; Kondorosi et al., 2000; Serapiglia et al., 2015; Iannicelli et al., 2020). Shang et al. (2020) found that ploidy notably affected the stomatal traits of *Populus*, but did not affect the serration number on the leaf margin and petiole length of the leaf. Triploid elite plants in white poplar hybrids showed better wood properties and fiber traits compared with diploid control (Kang, 2016). In *Eucalyptus grandis* × *E. urophylla*, triploid and tetraploid plants presented wider trunks, taller trees with longer stems, wider crowns, and higher BD compared with diploid plants (Longui et al., 2021). In our study, the diploid and triploid groups differed significantly in FL. The significant difference of FL/W between diploids and triploids could be attributed to a consequence of collinearity between FL and FL/W, because there was no significant difference in FW between the ploidy levels. Further analyses of variance components showed that ploidy contributed to the variation of FL and FL/W at 77 and 50%, respectively, indicating the huge potential of triploid breeding for the improvement of fiber traits in *Populus*.

The utilization of heterosis is an important topic of plant breeding. Compared with the selfing crops, hybridization breeding of the tree species usually results in extensive allelic segregation and assortment in the progeny due to the high heterozygosity of the parents. Generally, wood property and fiber traits vary with genotype in tree species (Zobel and van Buijtenen, 1989). Pliura et al. (2007) found significant genotypic variation in the wood density and growth traits of poplar hybrids. A remarkable difference of FL from wood disks at 1.5-m height was detected among genotypes in *Populus* (DeBell et al., 2002). In triploid white poplar, the genotype also resulted in significant differences in BD, FL, FW, and FL/W traits (*p* < 0.001, Wu et al., 2013). In our study, it was found that the wood and fiber traits varied significantly among the genotypes. The contribution of the genotype effect to the measured wood and fiber traits ranged from 7 to 62% (Figure 5). For the BD, LC, and FW traits, the genotype effect was the dominant source of their variation. Therefore, no matter in cross-breeding or triploid breeding of *Populus*, genotype screening in progeny based on the multiple parental combinations is important for the improvement of wood property and fiber traits.

In addition, gender dimorphism is an important evolutionary transition in many plant families (Miller and Venable, 2000). For dioecious plants, gender dimorphism may lead to phenotypic divergence. In *Silene latifolia*, the male plants had significantly wider calyx than the female plants (Yu et al., 2011). Maldonado-López et al. (2014) found that the female plants of *Spondias purpurea* showed a higher plant size and nutritional quality than the male plants. In a collected natural population of *P. tomentosa*, both FL and FW of the female group were significantly larger than those of the male group, but there were no significant differences between the female and male groups in wood chemical compositions (Du et al., 2014). In our study, however, no statistical difference was detected between the female and male groups in any measured BD, LC, and fiber traits within the diploid or triploid groups. The analyses of variance components showed that gender explained 23% of the variation, but rarely contributed to the variation of the LC, FL, FW, and FL/W traits. The different effects of gender dimorphism between our study and the earlier ones might be attributed to the differences in species and population for investigation. Recently, male varieties of *Populus* were recommended for plantation in China, because fluff catkins of female plants may cause severe environmental pollution in rural and urban areas, even potential fire risk (Janković et al., 2021). The minor effects of gender on wood properties suggest that the selection of male genotypes might not reduce the pulp-industrial value of *Populus* in our experimental family.

An understanding of the relationships between these wood properties is of value in the development of a breeding program for the pulp and paper industry. In our study, both phenotypic and BLUP correlations between the traits were analyzed. The results showed that the phenotypic correlations had similar changes with the BLUP correlations, though the significance levels in several trait pairs varied, which reflected on the difference between the phenotypic value and BLUP-adjusted genotypic value. BLUP, presented by Henderson (1975), is a traditional method for predicting genetic parameters and has been commonly used in plant breeding (Resende, 2016). The BLUP method has good predictive accuracy for genetic parameters when the sample sizes are variable (Piepho et al., 2008). Compared with the phenotypic correlation, BLUP reflects the genetic correlation between traits, which can provide a better understanding of how traits are interrelated. In triploids, BD was significantly positively correlated with FL in BLUPs (*r* = 0.65, *p* = 0.030), suggesting that BD could be improved together with FL during triploid breeding. In the triploids of *P. tomentosa*, a significantly positive genotypic correlation between BD and FL was also found with the correlation coefficient ranging from 0.41 to 0.98 at different sites (Wu et al., 2013). There was a moderately negative BLUP correlation between LC and FL in the triploid group (*r* = −0.46, *p* = 0.159), in our study, suggesting that the selection of FL trait might lead to the decrease of LC in triploid breeding.

In this study, the CV_G_ for each measured trait was low, ranging from 4 to 11%, which indicates that the genetic variation is quite small for these wood traits. It means that changes from direct selection and correlated responses will be quite small for these traits in the family of our study. The CV_G_ values in triploids were no larger than that of the diploids in this family. In the triploids of *P. tomentosa*, the CV_G_ values of the wood and fiber traits were also very small, ranging from 1.4 to 6.4 at different sites (Wu et al., 2013). Therefore, to improve the wood and fiber traits, it is necessary to produce many hybrids and triploids for selection. To produce triploids of *Populus*, various methods for sexual polyploidization, such as 2n pollen induction, 2n female gamete induction, and crossing diploids with tetraploids, have been developed (Einspahr, 1984; Kang et al., 2000; Wang et al., 2010, 2012a, b; Li et al., 2019). The approach of 2n female gamete induction could produce more than 60% triploids (Wang et al., 2010, 2012a, b; Lu et al., 2013), which should be used to increase the number of triploids in this family. Alternatively, other species could be introduced as parents to improve the wood and fiber traits. In our study, the parents are both Aigeiros–Tacamahaca intersection hybrids (the female parent ‘Zheyin3#’ is derived from *P. pseudo-simonii* × *P. nigra* and the male parent BJY was generated by crossing *P. nigra* var *italica* with *P. cathayana*), resulting in limited variations of the traits in progeny. In future, more species, such as *P. simonii*, *P. nigra*, and *P. deltoides*, could be used for parental selection with large combining ability, and triploid induction should be conducted using the parents.

## Data Availability Statement

The original contributions presented in the study are included in the article/supplementary material, further inquiries can be directed to the corresponding author/s.

## Author Contributions

JW conceived and designed the research. X-YH, JS, and Y-HZ collected wood disks and conducted laboratory experiments. JS and D-LL analyzed the data. L-JS managed the field trial. X-YH, D-LL, and JW wrote and revised the manuscript. All authors read and approved the manuscript.

## Conflict of Interest

The authors declare that the research was conducted in the absence of any commercial or financial relationships that could be construed as a potential conflict of interest.

## Publisher’s Note

All claims expressed in this article are solely those of the authors and do not necessarily represent those of their affiliated organizations, or those of the publisher, the editors and the reviewers. Any product that may be evaluated in this article, or claim that may be made by its manufacturer, is not guaranteed or endorsed by the publisher.
